# Chronic use of hydroxychloroquine did not protect against COVID-19 in a large cohort of patients with rheumatic diseases in Brazil

**DOI:** 10.1186/s42358-021-00217-0

**Published:** 2021-10-07

**Authors:** Gecilmara Salviato Pileggi, Gilda Aparecida Ferreira, Ana Paula Monteiro Gomides Reis, Edgard Torres Reis-Neto, Mirhelen Mendes Abreu, Cleandro Pires Albuquerque, Nafice Costa Araújo, Ana Beatriz Bacchiega, Dante Valdetaro Bianchi, Blanca Bica, Eloisa Duarte Bonfa, Eduardo Ferreira Borba, Danielle Christinne Soares Egypto Brito, Ângela Luzia Branco Pinto Duarte, Rafaela Cavalheiro Espírito Santo, Paula Reale Fernandes, Mariana Peixoto Guimarães, Kirla Wagner Poti Gomes, Adriana Maria Kakehasi, Evandro Mendes Klumb, Cristina Costa Duarte Lanna, Claudia Diniz Lopes Marques, Odirlei André Monticielo, Licia Maria Henrique Mota, Gabriela Araújo Munhoz, Eduardo Santos Paiva, Helena Lucia Alves Pereira, José Roberto Provenza, Sandra Lucia Euzébio Ribeiro, Laurindo Ferreira Rocha Junior, Camila Santana Justo Cintra Sampaio, Vanderson Souza Sampaio, Emília Inoue Sato, Thelma Skare, Viviane Angelina de Souza, Valeria Valim, Marcus Vinícius Guimarães Lacerda, Ricardo Machado Xavier, Marcelo Medeiros Pinheiro

**Affiliations:** 1 Rheumatology Unit of Escola Paulista de Medicina (Unifesp/ EPM), São Paulo, SP Brazil; 2 Hospital das Clínicas, Universidade Federal de Minas Gerais (UFMG), Belo Horizonte, MG Brazil; 3 Hospital Universitário de Brasília da Universidade de Brasília, EBSERH (HUB-UnB), Brasília, DF Brazil; 4 Hospital São Paulo da Universidade Federal de São Paulo, Escola Paulista de Medicina (Unifesp/ EPM), São Paulo, SP Brazil; 5 Hospital Universitário Clementino Fraga Filho, Universidade Federal Do Rio de Janeiro (UFRJ), Rio de Janeiro, RJ Brazil; 6 Hospital Do Servidor Público Estadual, Instituto de Assistência Médica Ao Servidor Público Estadual (IAMSPE), São Paulo, SP Brazil; 7 Santa Casa de Misericórdia Do Rio de Janeiro (HGSCMRJ), Rio de Janeiro, RJ Brazil; 8 Rheumatology Division, Hospital das Clínicas HCFMUSP, Faculdade de Medicina, Universidade de São Paulo, São Paulo, SP Brazil; 9 Hospital Universitário Lauro Wanderley, Universidade Federal da Paraíba (UFPB), João Pessoa, PB Brazil; 10 Hospital das Clínicas da Universidade Federal de Pernambuco (UFPE), Recife, PE Brazil; 11 Hospital de Clínicas de Porto Alegre, Universidade Federal Do Rio Grande Do Sul (UFRGS), Porto Alegre, RS Brazil; 12 Hospital Universitário da Universidade Federal de Juiz de Fora (UFJF), Juiz de Fora, MG Brazil; 13 Santa Casa de Misericórdia de Belo Horizonte, Belo Horizonte, MG Brazil; 14 Hospital Geral de Fortaleza (HGF), Fortaleza, CE Brazil; 15 Hospital Universitário Pedro Ernesto, Universidade Do Estado Do Rio de Janeiro (UERJ), Rio de Janeiro, RJ Brazil; 16 Irmandade da Santa Casa de Misericórdia de São Paulo (ISCMSP), São Paulo, SP Brazil; 17 Universidade Federal Do Paraná (UFPR), Curitiba, PR Brazil; 18 Hospital Universitário Getúlio Vargas Universidade Federal Do Amazonas, Manaus, AM Brazil; 19 Pontifícia Universidade Católica de Campinas (PUC-CAMP), Campinas, SP Brazil; 20 Instituto de Medicina Integral Professor Fernando Figueira (IMIP/ PE), Recife, PE Brazil; 21 Instituto de Ensino E Pesquisa No Sírio Libanês, São Paulo, SP Brazil; 22 Fundação de Vigilância Em Saúde Do Amazonas, Manaus, AM Brazil; 23 Instituto Leônidas and Maria Deane, Fiocruz, Manaus, AM Brazil; 24 Fundação de Medicina Tropical Dr. Heitor Vieira Dourado, Manaus, AM Brazil; 25 Hospital Universitário Evangélico Mackenzie (HUEM), Curitiba, PR Brazil; 26 Hospital Universitário Cassiano Antonio de Moraes, Universidade Federal Do Espírito Santo, Vitória, ES Brazil

**Keywords:** COVID-19, Hydroxycloroquine, Rheumatic diseases

## Abstract

**Background:**

There is a lack of information on the role of chronic use of hydroxychloroquine during the SARS-CoV-2 outbreak. Our aim was to compare the occurrence of COVID-19 between rheumatic disease patients on hydroxychloroquine with individuals from the same household not taking the drug during the first 8 weeks of community viral transmission in Brazil.

**Methods:**

This baseline cross-sectional analysis is part of a 24-week observational multi-center study involving 22 Brazilian academic outpatient centers. All information regarding COVID-19 symptoms, epidemiological, clinical, and demographic data were recorded on a specific web-based platform using telephone calls from physicians and medical students. COVID-19 was defined according to the Brazilian Ministry of Health (BMH) criteria. Mann–Whitney, Chi-square and Exact Fisher tests were used for statistical analysis and two binary Final Logistic Regression Model by Wald test were developed using a backward-stepwise method for the presence of COVID-19.

**Results:**

From March 29th to May 17st, 2020, a total of 10,443 participants were enrolled, including 5166 (53.9%) rheumatic disease patients, of whom 82.5% had systemic erythematosus lupus, 7.8% rheumatoid arthritis, 3.7% Sjögren’s syndrome and 0.8% systemic sclerosis. In total, 1822 (19.1%) participants reported flu symptoms within the 30 days prior to enrollment, of which 3.1% fulfilled the BMH criteria, but with no significant difference between rheumatic disease patients (4.03%) and controls (3.25%). After adjustments for multiple confounders, the main risk factor significantly associated with a COVID-19 diagnosis was lung disease (OR 1.63; 95% CI 1.03–2.58); and for rheumatic disease patients were diagnosis of systemic sclerosis (OR 2.8; 95% CI 1.19–6.63) and glucocorticoids above 10 mg/ day (OR 2.05; 95% CI 1.31–3.19). In addition, a recent influenza vaccination had a protective effect (OR 0.674; 95% CI 0.46–0.98).

**Conclusion:**

Patients with rheumatic disease on hydroxychloroquine presented a similar occurrence of COVID-19 to household cohabitants, suggesting a lack of any protective role against SARS-CoV-2 infection.

*Trial registration* Brazilian Registry of Clinical Trials (ReBEC; RBR – 9KTWX6).

## Background

Severe Acute Respiratory Syndrome Coronavirus (SARS-CoV-2) is the etiological agent of COVID-19, a public health emergency with relevant challenges worldwide and different epidemic curves and mortality rates between countries [1, 2]. The disease has a heterogeneous clinical spectrum, from asymptomatic forms to severe systemic involvement, including pneumonia, cytokine storm syndrome, endotheliocyte damage, and thrombotic events [3–8].

Initial data have suggested that SARS-CoV-2 does not appear to cause more serious disease in immunosuppressed patients [9–11] and this clinical observation has drawn attention to a potential beneficial or ‘protective’ effect of medications used to control rheumatic diseases (RD) [12–15].

Chloroquine (CQ) and hydroxychloroquine (HCQ), immunomodulator drugs traditionally used to treat malaria and rheumatic diseases (RD), such as rheumatoid arthritis (RA), systemic lupus erythematosus (SLE), and primary Sjögren syndrome (pSS) [16–18], were pointed out as effective pharmacological strategies against COVID-19 in vitro and in anecdotal reports [19–21]. In addition, it could attenuate the cytokine storm observed in moderate or severe COVID-19 forms mitigating unfavorable outcomes. However, there are controversial data regarding their efficacy and safety to treat COVID-19 patients and a recent randomized controlled trial did not show any beneficial effect in patients hospitalized with mild-to-moderate disease when compared to standard care [22–27]. Gentry et al. did not found any significant difference regarding the incidence of active SARS-CoV-2 infection between patients with rheumatic diseases receiving hydroxychloroquine and patients without it [28].

## Methods

### Study design and participants

This study aimed to evaluate the frequency of COVID-19 in patients with RD in HCQ, in comparison with their cohabitants during the SARS-CoV-2 pandemic in Brazil. This is a cross-sectional, observational, paired study, including adult volunteers (≥ 18 years of age), with a known previous diagnosis of RD, using HCQ for at least 30 days before the initial consultation. According to the previously defined classification criteria, the cohort included patients with SLE [29]; RA [30]; pSS [31]; systemic sclerosis [32]; inflammatory myopathies [33]; mixed connective tissue disease [34]; hand osteoarthritis [35, 36], and chikungunya-related arthropathy [37].

Household cohabitants aged over 18, without RD and not using antimalarials for any purpose, were chosen as the control group to ensure more homogeneous environmental exposure to the SARS-CoV-2 infection among participants during the community viral transmission, instead of including rheumatic disease patients not using antimalarials, who would probably present a different set of diseases and different epidemiological exposure.

All participants with a history of solid organ or bone marrow transplantation, neoplasm in the previous 12 months, immunoglobulin use in the previous 30 days, current kidney replacement therapy, thymus disease, other immunodeficiencies, or positive HIV status were excluded.

Twenty-two tertiary rheumatology centers, representing the five geographic regions of Brazil and thus encompassing most of the population variability, joined the task-force study. The inclusion period was the first 8 weeks of community transmission in Brazil. This manuscript is part of a larger prospective study with 24-week follow-up.

### Procedures

Participants were enrolled in this multi-center study and included through phone calls performed by previously trained medical students and physicians. Details were obtained of epidemiological and demographic data, as well as RD status and current treatment data. In addition, specific information about the COVID-19 symptoms, hospitalization, need for intensive care, and death was recorded in both groups and represents the main endpoints of this cohort. All the data are stored and managed using an electronic on-line platform (REDCap).

Patients taking other dosages of HCQ than 5 mg/kg/day (maximum 400 mg/ day) or using CQ were not included in the final analysis.

### Outcomes

The results presented in this manuscript are from a cross-sectional database analysis at baseline (first telephone interview-inclusion visit) with the main outcome being the occurrence of COVID-19, according to the Brazilian Ministry of Health (BMH), within 30 days prior to enrollment [38]. Confirmatory tests have not been routinely performed in Brazil for patients with mild symptoms of SARS-CoV-2 infection, only for moderate-severe cases.

### Outcome definitions

**Fig. 1:**
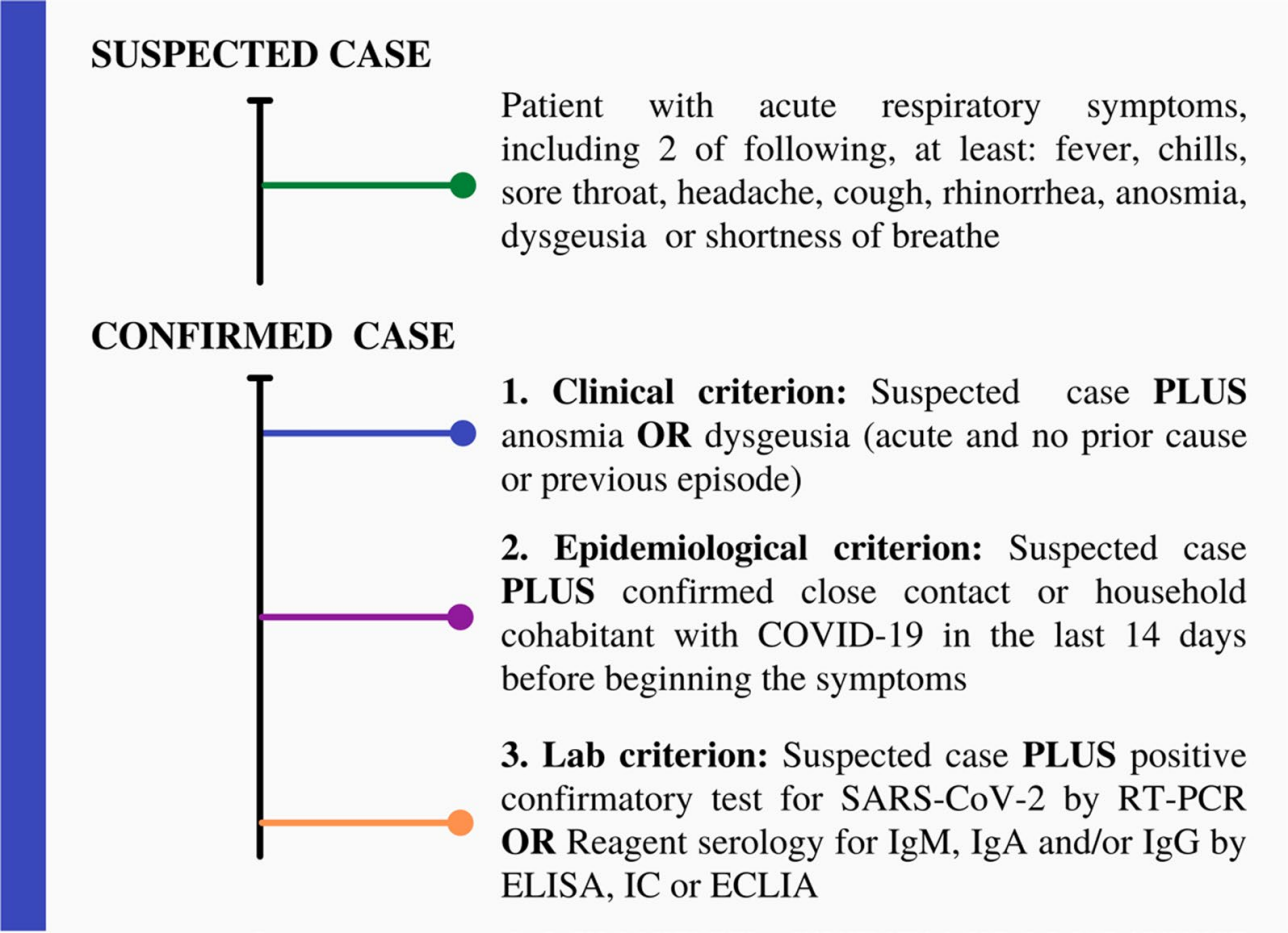
Outcome definition: participants were classified according to the Brazilian Ministry of Health (BMH) criteria using the definition for COVID-19. Individuals with more than 3 days of influenza-like illness symptoms were considered for this analysis

Participants in this study were defined with COVID-19, according to the most recent criteria established by the Brazilian Ministry of Health (BMH) during the pandemic period. The BMH criteria was applied to symptomatic patients based on the clinical, epidemiological and laboratory criteria, were considered as COVID-19 (Fig. 1).

### Statistical analyses

Descriptive statistics were expressed as mean, standard deviation, as well as frequency (%) and difference 95% confidence intervals (95% CI). The Kolmogorov–Smirnov test was used to verify a normal data distribution. Two binary Final Logistic Regression Model by Wald test were developed using a backward-stepwise method for the presence of COVID-19, including Odds Ratio (OR) and their respective 95% CI. The first model considered both groups (cases and controls) and was adjusted for age, sex, lung and kidney disease, hypertension, diabetes, and influenza vaccine within the previous 30 days. The second one included only RD patients, adjusted for lung disease, corticosteroids, systemic sclerosis, and influenza vaccine within the previous 30 days. Only variables with p value below 0.2 found in the first model was added to the second model. A *P*-value under 0.05 was considered significant. The statistical analysis was performed using IBM-SPSS v.20.0 software.

## Results

**Fig. 2:**
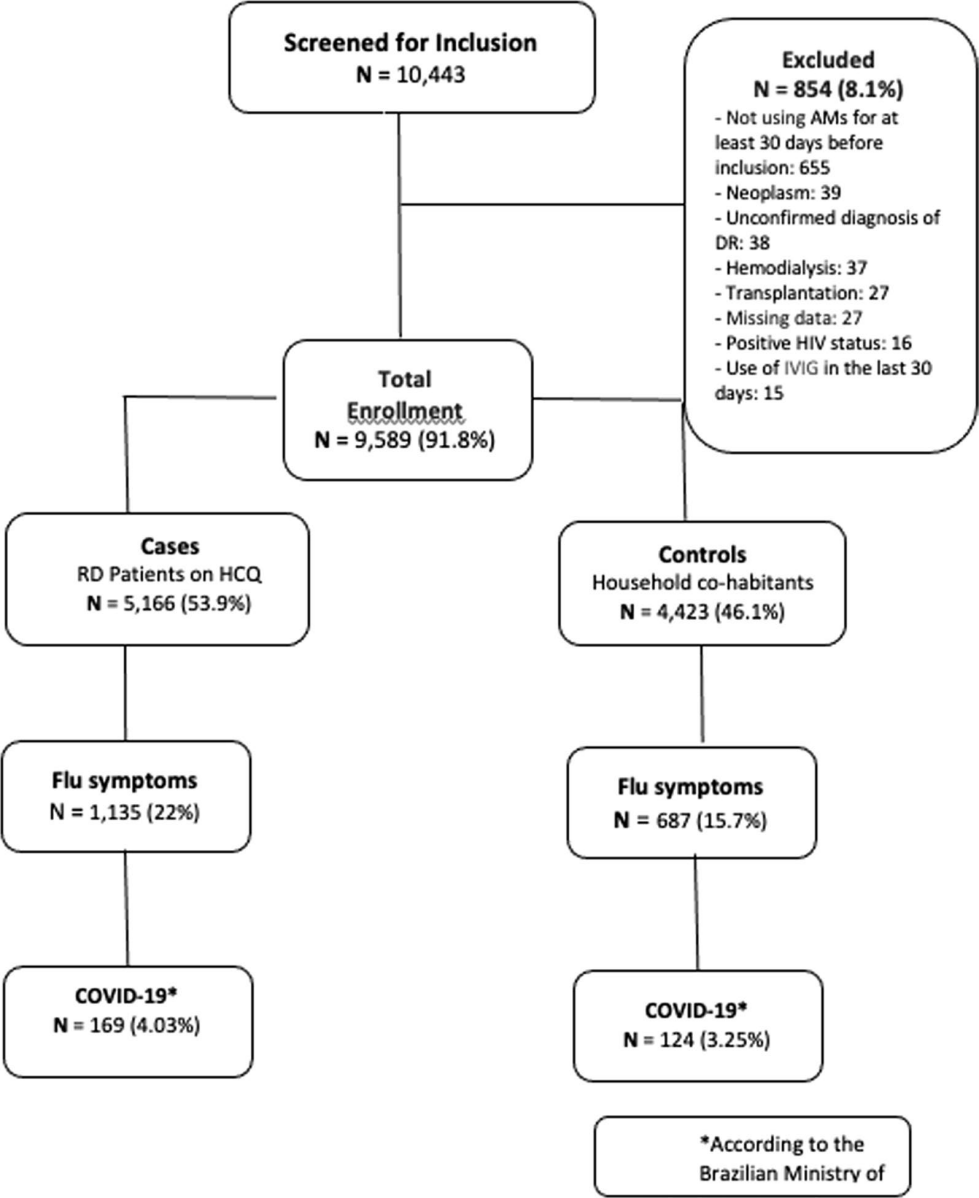
Flowchart of participants enrolled in this study, considering influenza-like illness symptoms and a diagnosis of COVID-19, according to the Brazilian Ministry of Health criteria

From March 29th to May 17th, 2020 (8-week period), a total of 9589 participants from 97 Brazilian cities were enrolled at baseline, including 5166 (53.9%) patients with RD on HCQ (5 mg/kg/day, maximum dosage of 400 mg), and 4423 (46.1%) cohabitants living in the same household. Of these, 854 (8.1%) individuals were excluded according to the eligibility criteria (Fig. 2).

Although statistically different, the difference between the mean age and frequency of contact with a confirmed case of COVID-19 were not clinically relevant. There was a higher frequency of females in the patients’ group and a higher frequency of males in the household cohabitants. All concomitant diseases evaluated were significantly more common in RD patients than the control group, except for diabetes. On the other hand, social distancing and influenza vaccination were reported more frequently by RD patients (Table 1).

**Table 1:** Epidemiological and clinical data between patients with rheumatic diseases and household contacts at baseline

Variables	All	RD patients	Household co-habitants	Difference (CI 95%)	p*
	N = 9589	N = 5166	N = 4423		
	n (%)	n (%)	n (%)		
Age, years (SD)	43.5 (14.9)	43.1 (13.9)	44.0 (16.1)	0.90 (0.29; 1.50)	**0.039**
*Sex*					
Women	6617 (69.4)	4772 (92.6)	1845 (42.2)	50.4 (48.8; 52.0)	** < 0.001**
Men	2912 (30.6)	382 (7.4)	2530 (57.8)		
*Schooling*					
Basic or illiterate	2522 (26.5)	1296 (25.1)	1226 (28)	2.9 (1.1; 4.7)	** < 0.001**
High school	4027 (42.2)	2166 (42)	1861 (42.6)	0.6 (− 1.39; 2.6)	
College	2983 (31.3)	1697 (32.9)	1286 (29.4)	3.5 (1.6; 5.4)	
*Profession*					
Customer assistance	1911 (20.2)	946 (18.5)	965 (22.2)	3.7 (2.1; 5.3)	** < 0.001**
Healthcare	683 (7.2)	443 (8.7)	240 (5.5)	3.2 (2.2; 4.2)	
Safety professionals	182 (1.9)	43 (0.8)	139 (3.2)	2.4 (1.8; 3.0)	
Education	636 (6.7)	438 (8.6)	198 (4.6)	4.0 (3.0; 4.9)	
Housewife	1662 (17.6)	1236 (24.2)	426 (9.8)	14.4 (12.9; 15.9)	
Others	4382 (46.3)	2011 (39.3)	2371 (54.6)	15.3 (13.3; 17.3)	
*Contact with confirmed case of COVID-19*					
No	8136 (85.3)	4484 (86.9)	3652 (83.4)	3.5 (2.1; 4.9)	** < 0.001**
Yes	727 (7.6)	380 (7.4)	347 (7.9)	0.5 (− 0.6; 1.6)	
Unknown	673 (7.1)	294 (5.7)	379 (8.7)	3.0 (1.9; 4.1)	
Family unit in social distancing	5787 (60.7)	3235 (62.7)	2552 (58.4)	4.3 (2.3; 6.3)	** < 0.001**
Heart disease	496 (5.3)	314 (6.2)	182 (4.3)	1.9 (1.0; 2.8)	** < 0.001**
Diabetes	703 (7.5)	339 (6.7)	364 (8.5)	1.8 (0.7; 2.9)	** < 0.001**
Lung disease	497 (5.3)	357 (7)	140 (3.3)	3.7 (2.8; 4;6)	** < 0.001**
Kidney disease	602 (6.4)	565 (11.1)	37 (0.9)	10.2 (9.3; 11.1)	** < 0.001**
Hypertension	2673 (28.6)	1692 (33.3)	981 (23)	10.3 (8.5; 12.1)	** < 0.001**
Influenza vaccine within last 30 days	2584 (27.2)	1527 (29.6)	1057 (24.2)	5.4 (3.6; 7.2)	** < 0.001**

Bold values indicate statistical significance (p < 0.05)

The results are expressed as means, standard deviation and percentages

CI, confidence interval; RD, rheumatic diseases; COVID-19, Coronavirus disease 2019

^*^Chi-square test

Most of the RD patients had SLE (N = 4243; 82.5%), followed by RA (N = 402; 7.8%), and pSS (N = 192; 3.7%). Among the 5166 RD patients, 97.5% are using HCQ, of whom 522 (10.1%) take it as monotherapy and 4644 (89.9%) combined with other therapies, such as corticosteroids (37.0%) and immunosuppressant drugs (48.9%). The remaining 2.5% from antimalarials users were taking other chloroquine salts, particularly diphosphate, and they were excluded from this final analysis (Table 2).

**Table 2:** Main rheumatic diseases and concomitant medication at baseline

	N (%)
*Rheumatic disease*	
Systemic lupus erythematous	4243 (82.5)
Rheumatoid arthritis	402 (7.8)
Primary Sjögren syndrome	192 (3.7)
Mixed connective tissue disease	75 (1.5)
Osteoarthritis	66 (1.3)
Systemic sclerosis	43 (0.8)
Inflammatory myopathies	34 (0.7)
Chikungunya	18 (0.4)
Other	69 (1.3)
*Antimalarials*	
Hydroxychloroquine (HCQ)	5035 (97.5)
HCQ use time (years), mean (SD)	7.2 (6.2)
Chloroquine diphosphate (CD)	131 (2.5)
CD use time (years), mean (SD)	10.6 (7.4)
*Concomitant medication related to RD*	
Glucocorticoids	1895 (36.7)
< 10 mg/day	1394 (73.6)
≥ 10 mg/day	462 (24.4)
Ibuprofen	35 (0.7)
IV Methylprednisolone (pulse)	30 (0.6)
Cyclophosphamide (oral and pulse)	73 (1.4)
Synthetic conventional DMARDs	2444 (47.3)
Methotrexate	631 (12.2)
Sulfasalazine	16 (0.3)
Azathioprine	983 (19.0)
Leflunomide	96 (1.9)
Cyclosporine	80 (1.5)
Mycophenolate mofetil	657 (12.7)
Biological or target-specific DMARDs	181 (3.5)
TNF inhibitors	17 (0.3)
Belimumab	52 (1.0)
Rituximab	81 (1.6)
Abatacept	17 (0.3)
Tocilizumab	7 (0.1)
Tofacitinib	7 (0.1)

The results are expressed as means, standard deviation and percentages; DMARDs, disease activity-modifying drugs; some individuals are taking more than one synthetic DMARD

In total, 1822 (19.1%) participants reported influenza-like illness symptoms within the 30 days prior to enrollment, of whom 293 (3.1%) individuals fulfilled the BMH criteria for a COVID-19 diagnosis [38]. In general, the frequency of self-reported influenza-like illness symptoms was significantly higher in RD patients, including those with severe symptoms (such as shortness of breath), except fever and anosmia (Table 3).

**Table 3:** Self-reported influenza-like illness symptoms and a COVID-19 diagnosis in patients with rheumatic diseases and household contacts at baseline

Symptoms	All N = 9589 n (%)	RD Patients N = 5164 n (%)**	Household co-habitants N = 4378 n (%) *	Difference (95% CI)	p*
Any	1822 (19.1)	1135 (22)	687 (15.7)	6.3 (4.7; 7.9)	** < 0.001**
Fatigue	531 (5.6)	328 (6.4)	203 (4.6)	1.8 (0.9; 2.7)	** < 0.001**
Headache	734 (7.7)	453 (8.8)	281 (6.4)	2.4 (1.3; 3.5)	** < 0.001**
Rhinorrhea	976 (10.2)	601 (11.6)	375 (8.6)	3.0 (1.8; 4.2)	** < 0.001**
Dysgeusia	242 (2.5)	146 (2.8)	96 (2.2)	0.6 (0; 1.2)	**0.049**
Shortness of breath	266 (2.8)	188 (3.6)	78 (1.8)	1.8 (1.2; 2.4)	** < 0.001**
Sore throat	704 (7.4)	455 (8.8)	249 (5.7)	3.1 (2.1; 4.1)	** < 0.001**
Fever	486 (5.1)	276 (5.3)	210 (4.8)	0.5 (− 0.4; 1.4)	0.225
Anosmia	209 (2.2)	120 (2.3)	89 (2)	0.3 (− 0.3; 0.9)	0.333
Cough	910 (9.5)	579 (11.2)	331 (7.6)	3.6 (2.4; 4.8)	** < 0.001**
Fever AND Shortness of breath	123 (1.3)	80 (1.9)	43 (1.2)	0.7 (0.2; 1,2)	**0.005**
Fever AND Cough AND Shortness of breath	83 (0.9)	53 (1.3)	30 (0.8)	0.5 (0.1; 0.9)	**0.034**
BMH COVID-19 criteria	293 (3.1)	169 (4.03%)	124 (3.25%)	0.78 (− 0.05; 1.60)	**0.065**

Bold values indicate statistical significance (p < 0.05)

The results are expressed as means, standard deviation and percentages

BMH, Brazilian Ministry of Health

^*^There are 45 missing data; **There are 2 missing

Considering a COVID-19 diagnosis, there was no significant difference in the number of cases between RD patients (4.03%) and the control group (3.25%) (OR 0.78, − 0.05; 1.60). Men (OR 0.71; 95% CI 0.52–0.98, p = 0.043) participants had lower likely of having the disease. On the other hand, individuals with previous lung disease (OR 1.63; 95% CI 1.03–2.58, p = 0.038) were more likely to present clinically confirmed COVID-19 in the final logistic regression model, after adjustments for multiple confounders, using the variables with p < 0.2 in the first (Table 4).

**Table 4:** Final logistic regression model considering all individuals enrolled at baseline

Variables	Binary analysis			Multivariate analysis	
	No symptoms N = 7720	Clinically Confirmed COVID-19 N = 293	p	OR (95% CI)	P***
	n (%)	n (%)			
Age (y), mean (SD); med. (min–max.)	43.9 (15.2); 42 (18–98)	41.6 (13.0); 41 (18–90)	0.028*	0.989 (0.981; 0.997)	**0.008**
*Group*					
Household cohabitants	3691 (47.8)	124 (42.3)	0.065**	1	-
RD patients	4029 (52.2)	169 (57.7)		1.10 (0.83; 1.46)	0.526
*Sex*					
Women	5259 (68.2)	218 (75.4)	0.01**	1	-
Men	2450 (31.8)	71 (24.6)		0.71 (0.52; 0.98)	**0.043**
*Schooling*					
Basic or illiterate	2110 (27.4)	64 (21.8)	0.091**		
High school	3280 (42.6)	139 (47.4)			
College	2317 (30.1)	90 (30.7)			
Family in social distancing	4728 (61.3)	172 (58.9)	0.402**		
Heart disease	398 (5.3)	17 (6)	0.570**		
Diabetes	585 (7.7)	17 (6)	0.292**		
Lung disease	367 (4.8)	21 (7.4)	0.048**	1.63 (1.03; 2.58)	**0.038**
Kidney disease	465 (6.1)	23 (8.2)	0.169**		
Hypertension	2165 (28.6)	78 (27.7)	0.730**		
Influenza vaccine within 30 last days	2138 (27.8)	63 (21.6)	0.022**		

Bold values indicate statistical significance (p < 0.05)

Outcome is clinically confirmed COVID-19 diagnosis

Y, years; SD, standard deviation; med., median; min., minimum; max., maximum

^*^Mann–Whitney test; **Chi-square test; ***Wald test by final logistic regression model

Considering only RD patients, having systemic sclerosis and current use of glucocorticoids (daily dosage above 10 mg) had a harmful effect for a COVID-19 diagnosis while a recent influenza vaccination had a protective role (OR 0.674; 95% CI 0.46–0.98), after multiple adjustments for sex, age, concomitant medication, immunosuppressant drugs, and comorbidities, regardless of chronic HCQ use, (Table 5).

**Table 5:** Final logistic regression model regarding rheumatic disease patients enrolled at baseline

Variables	Binary analyses			Multivariate analyses	
	No symptoms N = 4029 n (%)	Clinical Covid-19 N = 169 n (%)	p	OR (95% CI)	P****
Influenza vaccine within last 30 days	1235 (30.7)	39 (23.1)	0.034**	0.676 (0.465; 0.984)	**0.041**
IV Methylprednisolone (pulse)	21 (0.5)	2 (1.2)	0.236***		
*Glucocorticoids*					
No	2555 (63.9)	97 (58.1)	0.004**	1	-
< 10 mg/day	1099 (27.5)	43 (25.7)		0.965 (0.662; 1.41)	0.854
> = 10 mg/day	343 (8.6)	27 (16.2)		2.07 (1.33; 3.22)	**0.001**
scDMARDs	1875 (46.5)	84 (49.7)	0.419**		
Biological or tsDMARDs	120 (3)	7 (4.1)	0.387**		
RA	317 (7.9)	16 (9.5)	0.466**		
MCTD	56 (1.4)	2 (1.2)	> 0.99***		
SS	39 (0.9)	4 (2.4)	0.042***	3.81 (1.31; 11.05)	**0.014**
SLE	3304 (82)	134 (79.3)	0.414**		
IM	26 (0.6)	1 (0.6)	0.703***		
OA	60 (1.5)	1 (0.6)	0.518***		
pSjS	150 (3.79)	6 (3.6)	0.560***		
Another RD	55 (1.4)	4 (2.4)	0.299***		

Bold values indicate statistical significance (p < 0.05)

RA, Rheumatoid arthritis; SLE, Systemic lupus erythematous; RD, Rheumatic diseases; MCTD, Mixed connective tissue disease; SS, Systemic sclerosis; IM, Inflammatory myopathies; OA, Osteoarthritis; pSjS, Primary Sjögren syndrome; sc, synthetic conventional; ts, target-specific; DMARDs, Disease Activity-Modifying Drugs; Model 3, Outcome is COVID-19 diagnosis, according to the Brazilian Ministry of Health criteria; y, years; SD, standard deviation; med., median; min., minimum; max., maximum

^*^Mann–Whitney test; **Chi-square test; ***Fischer’s exact test; ****Wald test by final logistic regression model

## Discussion

Our results showed patients with RD on HCQ had a similar likelihood of presenting a COVID-19 diagnosis, according to the BMH criteria, when compared to cohabitants living in the same household during the first 8 weeks of community transmission in Brazil. Considering that according to recent studies [12, 39], patients with RD present a similar incidence of COVID-19 to the general population but with a potentially more unfavorable outcome [40, 41] and higher mortality rate [42, 43], we were not able to confirm our preliminary hypothesis in demonstrating a potential beneficial effect of chronic HCQ use against SARS-CoV-2 [44] in a population that traditionally has a higher prevalence of respiratory diseases.

Moreover, our data showed a higher frequency of influenza-like illness symptoms, including those with greater severity, especially shortness of breath, in patients with RD when compared with controls, suggesting these individuals should maintain social distancing, especially those that work with customer assistance, such as healthcare, teaching, and safety professionals [12, 45–50]. However, it is worth highlighting that patients with RD may report more symptoms than controls due to different behavior in relation to the perception of signs and symptoms because of the information they receive about their underlying disease from healthcare professionals and the combination of disease activity, as well as that the immunosuppression may predispose them to more infectious diseases that cause influenza-like illness symptoms such as influenza, adenovirus, and others [51].

Although CQ has in vitro activity against influenza, HCQ use did not prevent infection or decrease the risk of influenza infection [52–57]. Thus, our data are supported by current evidence demonstrating a lack of association between HCQ and COVID-19 considering pre-exposure (PrEP) and post-exposure prophylaxis especially in individuals at risk, such as healthcare professionals, as well as more recent randomized clinical trials, including mild-moderate and severe forms of SARS-CoV-2 infection [22, 24, 26, 27, 58–62].

In our total sample, men had a lower risk of COVID-19 than women (OR 0.71; 95% CI 0.52–0.98). This aspect could be related to higher frequency of female in patients group than in the control group because of inclusion approach that prioritized household contact paired for age (husband and wife more frequently). Also, men participants had less comorbidities and used less glucocorticosteroids. The current literature has shown a similar incidence between men and women, but with a poorer outcome in the former [1, 63–65].

Patients with RD using a daily GC dosage above 10 mg/day (prednisone equivalent), particularly above 20 mg/day, presented a two times higher risk of COVID-19 in our cohort. These data confirm previous findings showing a harmful effect of GC on the infection rate in immune-mediated RD patients, especially lupus [66], hampering the immune response against several infectious agents, including SARS-CoV-2 [67–70]. More recently, Gianfrancesco et al. also reported a higher risk of hospitalization in individuals using more than 10 mg/day (OR 2.05; 95% CI 1.06–3.96) and no significant association with HCQ, in agreement with our findings [41]. On the other hand, performing a sensitivity analysis excluding patients that received more than 10 mg/day of glucocorticoids from the RD group, and we observed quite similar findings (data not shown), suggesting that the risk for COVID-19 did not change when adjusted for corticosteroids (high vs. low dosage). It is important taking into consideration the low daily GC dosage (< 10 mg in almost 75% of them) and low proportion of current pulse therapy (around 2% of cyclophosphamide or methylprednisolone).

In the final multivariate model, systemic sclerosis was the only RD related to COVID-19, regardless of interstitial lung disease or the use of HCQ, as pointed out by some authors [71–73]. Nonetheless, an Italian phone interview study did not find any association regarding a higher risk in SS patients [39].

Interestingly, some of the main comorbidities associated with an unfavorable outcome and increased risk of death, such as diabetes, and heart and kidney diseases [1, 39, 64] were not significantly associated with COVID-19 in our patients with RD. In addition, the self-reporting of fever and/or anosmia, more specific symptoms of COVID-19, was also not different between RD patients and controls [74].

Although post influenza vaccine side effects could also have been a potential confounding factor, we found the influenza vaccine had an independent protective role in RD patients (OR 0.674; 95% CI 0.463–0.979), reducing the diagnosis of COVID-19 during the beginning of national vaccination campaign. Our data reinforce the effectiveness and safety of this approach in RD patients [75]. In addition, it is noteworthy pointing out this potential protective effect could be related to some bias, especially some behavioral attitudes (social distancing, strict masking and other self-care measurements) that are more observed in immunosuppressed patients.

To the best of our knowledge, this is the largest epidemiological study designed to evaluate the preventive role of HCQ to development of COVID-19 in patients with RD using HCQ. Some strengths should be considered, such as sampling size, the control group with the same epidemiological setting, weekly data quality monitoring, specific platform to collect all the information using serial, with national representation in pandemic times.

On the other hand, it is worth emphasizing some limitations of the study that are inherent to the COVID-19 pandemic, including the need for social distancing and specific guidance for the patients to avoid seeking medical care unless absolutely necessary. Therefore, in such a large population, we have only self-reported data, and a small number of confirmatory lab tests (RT-PCR and serology) and information on disease activity. The BMH criteria for COVID-19 have several similarities with the US criteria to define COVID-19 [76].

Another limitation was the lack of patients with RD not using HCQ as another control group. However, this approach could present other prescription biases, as SLE patients without antimalarial treatment are quite uncommon, except in those with previous toxicity (maculopathy, allergy, long-term remission, among others). The strategy of prioritizing and enrolling the household cohabitants was chosen because of the relevant epidemiological impact of COVID-19. A relevant clinical consideration is related to the severity of RD in the patients included in this cohort, since there were few patients taking biological DMARDs and cyclophosphamide. However, more recently, Zhong et al., in a Chinese retrospective study involving 6228 patients with autoimmune diseases that were enrolled in just 10 days and during sharp decline of COVID-19 outbreak in Hubei found lower risk of infection than patients taking other DMARDs (OR 0.09 [95% CI 0.01–0.94]; p = 0.044) [77].

As future perspectives, the shortage of HCQ with potential effects after withdrawal [78–81] will be further explored during the 24-week follow-up, as well as hospitalization and mortality rate [82].

## Conclusion

This study provides evidence of a non-protective role of chronic HCQ use (5 mg of the sulfate/kg/day) concerning uncomplicated COVID-19 in RD patients, regardless of comorbidities, immunosuppression therapy, and social distancing.

## Data Availability

The datasets used and/or analysed during the current study are available from the corresponding author on reasonable request.

## Abbreviations

BMH: Brazilian Ministry of Health
CI: Confidence intervals
CQ: Chloroquine
HCQ: Hydroxychloroquine
OR: Odds ratio
pSS: Primary Sjögren syndrome
RA: Rheumatic arthritis
RD: Rheumatic diseases
SLE: Systemic lupus erythematosus
